# 罕见肿瘤新药研发的思考

**DOI:** 10.3779/j.issn.1009-3419.2022.101.26

**Published:** 2022-07-20

**Authors:** 凌 唐, 媛媛 宋, 志敏 杨

**Affiliations:** 100022 北京，国家药品监督管理局药品审评中心 Center for Drug Evaluation, National Medical Products Administration, Beijing 100022, China

**Keywords:** 罕见肿瘤, 药物, 研发, Rare tumors, Drug, Research and development

## Abstract

近年来，我国抗肿瘤药物呈现持续增长趋势，我国抗肿瘤研发的活跃程度占全球比例增高。然而，进一步分析研发热点，抗肿瘤药物在各瘤种中的研发并不均衡。对于发病率低的罕见肿瘤，由于患者少，临床试验开展难度大，研发热情普遍较低。然而，患者的治疗需求，也会为制药企业带来潜在机遇。基础研究的发展，以及新的肿瘤分子分型的发现，使“罕见肿瘤”成为一种动态的概念，“罕见肿瘤”的范围可能随着治疗的精准化发展而逐步拓宽；也可能随着对肿瘤的认识逐步由组织细胞学向分子学层面发展，使某些伴有特定突变的瘤种，共同组合成一组非罕见的“泛肿瘤”。罕见肿瘤同时具备罕见病和肿瘤的特征。其药物研发既要满足肿瘤药物研发的要求，又要适应于罕见疾病药物研发的特点，因此药物研发中可参考罕见病药物的研发思路，同时结合肿瘤疾病的特征，充分利用非罕见瘤种临床试验中的数据，充分利用科学工具、精巧的试验设计，实现对罕见肿瘤药物研发的促进。本文将对罕见肿瘤领域新药审评中的思考进行总结，以期为业界提供参考。

## Abstract (en)

In recent years, China's anti-tumor drugs has shown a continuous growth trend, and the activity of anti-tumor research and development in China accounts for a higher proportion in the world. However, further analysis of research and development hotspots show that the research and development of anti-tumor drugs is uneven among different tumor types. Due to the small number of the patients, it is difficult to conduct clinical trials, resulting in less drug development in the field of rare tumors. However, patients' treatment needs will also bring potential opportunities for pharmaceutical companies. The development of basic research and the discovery of new molecular tumor typing make "rare tumors" a dynamic concept. The scope of "rare tumors" may gradually expand with the precise development of treatment; or as the knowledge of tumors gradually develops from histocytology to the molecular level, It is possible that certain tumors with specific mutations can be combined into a group of non-rare "pan-tumors". Rare tumors are characterized by both rare diseases and tumors. Its drug research and development should not only meet the requirements of tumor drug research and development, but also adapt to the characteristics of rare diseases. Therefore, in the drug research and development, we can refer to the research and development principle of rare disease drugs, combine with the characteristics of tumor diseases, make full use of non-rare tumor clinical trials, make full use of scientific tools and exquisite trial design, and realize the promotion of the research and development of rare tumor drugs. This paper will summarize the thoughts in the review of new drugs in the field of rare tumors, in order to provide guidance for the industry.

近年来，我国抗肿瘤药物呈现持续增长趋势，我国抗肿瘤研发的活跃程度占全球比例增高。根据clinicaltrial.com网站检索数据显示，2016年全球抗肿瘤药物临床试验累计为5, 535项，同期中国临床试验为1, 051项，占19%；而到2020年，全球临床试验累计为6, 162项，而中国为1, 579项，比例升高至25.6%。不仅如此，中国已越来越早期加入到全球同步研发中，国际多中心研究（Multicenter Randomized Clinical Trial, MRCT）的临床试验申请越来越多，其中不乏早期探索研究；中国与全球同步申报上市申请的数量逐渐增加；这些都是世界越来越信任中国临床试验、中国监管机构的表现。

快速发展的同时，我国抗肿瘤药物研发已不限于原有将进口产品“引进来”的模式，越来越多的国产药物“走出去”，中国制药企业开展MRCT数量逐渐增加，逐步经历本土化到国际化的转型，向世界分享与贡献中国智慧。

然而，进一步分析研发热点，抗肿瘤药物在各瘤种中的研发并不均衡。虽然随着中国制药企业的主导性增强，在我国高发瘤种，如肝癌、鼻咽癌、食管癌、胃癌等领域的研究显著增加，药物研发逐步向更符合中国患者治疗需求的方向发展，然而研发的热点依然是肺癌、乳腺癌等高发瘤种。对于发病率低的罕见肿瘤，由于患者少，临床试验开展难度大，研发热情普遍较低。本文将对罕见肿瘤领域新药审评中的思考进行总结，以期为业界提供参考。

## 1 罕见肿瘤药物研发趋势

罕见肿瘤有危及生命的特点，也是临床需求迫切的痛点，应该成为研发的关注点。无论是罕见病还是肿瘤，都是我国目前鼓励药物研发的方向。2015年国务院发布的《国务院关于改革药品医疗器械审评审批制度的意见》（国发[2015]44号）中指出：“加快审评审批防治艾滋病、恶性肿瘤、重大传染病、罕见病等疾病的创新药”；2018年国家药品监督管理局（National Medical Products Administration, NMPA）和国家卫生健康委员会联合发布的《关于优化药品注册审评审批有关事宜的公告》中也指出：“落实药品优先审评审批工作机制：对防治严重危及生命且尚无有效治疗手段疾病以及罕见病药品”。在政策的鼓励下，近些年，罕见肿瘤在逐步得到关注，越来越多的罕见肿瘤药物在中国开展临床试验，或申请上市。

胸膜间皮瘤是一种来源于胸膜间皮细胞的原发肿瘤，约占胸膜肿瘤的5%，是一种罕见的瘤种。在名为CheckMate-743（NCT02899299）的Ⅲ期临床研究^[1]^中证实，纳武利尤单抗注射液联合伊匹木单抗能够显著改善既往未经治疗的、不可切除的恶性胸膜间皮瘤患者的总生存期（overall survival, OS）。CheckMate-743研究最短随访22个月时，纳武利尤单抗联合伊匹木单抗降低患者死亡风险26%，患者的中位OS为18.1个月，而化疗组为14.1个月（HR=0.74, 95%CI: 0.60-0.91, *P*=0.002）。纳武利尤单抗联合伊匹木单抗组患者2年生存率为41%，而化疗组为27%。继2020年美国食品药品监督管理局（Food and Drug Administration, FDA）批准该联合治疗方案后，2021年6月NMPA也批准了纳武利尤单抗注射液联合伊匹木单抗注射液用于不可手术切除的、初治的非上皮样恶性胸膜间皮瘤成人患者。该联合方案成为了15年来首个为恶性胸膜间皮瘤患者带来生存获益的新系统性疗法。

再如神经纤维瘤，是一种由基因突变导致的神经系统遗传性肿瘤疾病，主要表现为全身多发的神经系统肿瘤。神经纤维瘤病发病率很低，属于罕见肿瘤。2020年4月，美国FDA宣布批准阿斯利康公司和默沙东公司共同开发的司美替尼（Selumetinib, AZD6244）上市，用于治疗2岁及2岁以上的1型神经纤维瘤病（neurofibromatosis type 1, NF1）儿童患者^[2]^，也是在该疾病中唯一一款治疗药物。目前司美替尼也在中国开展了临床试验。

在同步研发的大趋势下，中国罕见肿瘤与国际临床实践之间的差距在逐步缩小，国内外患者获得有效、更好治疗的时间差在逐步缩短。例如，美国FDA于2020年1月9日批准阿伐替尼用于携带*PDGFRA*外显子18突变（包括D842V突变）的不可切除或转移性胃肠道间质瘤成人患者^[3]^；2021年3月31日，阿伐替尼在中国的适应证正式获批。

## 2 打破对疾病的固有认识

罕见病通常指发病率“极低”的一组疾病。世界卫生组织（World Health Organization, WHO）将罕见病定义为患病人数占总人口的0.65‰-1‰的疾病或病变^[4]^。罕见病是一种相对的、动态变化的概念，随着人类对疾病的研究逐渐深入和新诊疗技术的不断发展，新的罕见病会被逐渐发现，而有些罕见病也可能演变为常见病^[5]^。上述动态的特征，在罕见肿瘤中更为典型。

### 2.1 从“非罕见”到“罕见”

罕见肿瘤是一种动态变化的概念。某些肿瘤发病率虽高，但其中伴有罕见突变、经治疗后难治复发的人群，则可能是罕见人群。从某种角度来说，罕见肿瘤的范畴随着对肿瘤的疾病认知进步、治疗的精准化发展而逐步拓宽，这在一定程度上为抗肿瘤药物的差异化研发带来机遇。

以弥漫大B型淋巴瘤（diffuse large B-cell lymphoma, DLBCL）为例，不同表型的DLBCL的临床表现、对治疗的反应和预后均有所不同，异质性明显。2000年就有研究^[6]^指出，生发中心B（germinal center B-cell, GCB）型DLBCL患者的预后优于非GCB（non-GCB）DLBCL。然而GCB DLBCL细胞表面缺乏固定B细胞受体（B cell receptor, BCR）集群，提示缺少慢性活化的BCR信号传导，可能导致布鲁顿的酪氨酸激酶（Bruton's tyrosine kinase, BTK）抑制剂对GCB DLBCL的疗效不佳。在伊布替尼单药治疗复发难治性DLBCL的1/2期前瞻性、开放标签、非随机临床研究中，non-GCB亚型客观缓解率（objective response rate, ORR）达到37%，GCB亚型ORR仅为5%。随后伊布替尼开展的Ⅲ期临床试验PHOENIX研究显示，在R-CHOP方案基础上增加伊布替尼并未改善non-GCB DLBCL研究人群的结局。这说明伊布替尼并不是对所有non-GCB亚型患者均有效，*DLBCL*的每一个表型之内仍存在异质性。该结果也提示以细胞起源的分类方式，并不能满足临床中分层治疗的需要。因此近年来，有研究对DLBCL进行了更为深入的研究，通过整合基因变异、基因拷贝数变异和基因易位与融合，将*DLBCL*的基因分型进一步完善。

2018年，Schmitz等^[7]^对574例DLBCL活检样本进行分析，发现4个显著的基因亚型，分别为MCD型（*MYD88L265P*突变和*CD79B*突变）、BN2型（*BCL6*融合和*NOTCH2*突变）、N1型（*NOTCH1*突变）和EZB型（*EZH2*突变和*BCL2*易位）。Wright等^[8]^在Schmitz分类的基础上，进一步分辨出ST2型、A53型和混合型。研究还发现MCD型是预后相对较差的DLBCL亚型，72.5%的MCD型DLBCL通过基因变异获得免疫逃逸。

MCD亚型DLBCL依赖B细胞受体信号的长期激活，其特征包括*CD79B*突变、活性升高、CD79A活性升高、BTKi活性显著升高及*MYD88*突变、活性升高，因此MCD亚型可能更适合应用BTKi治疗。基于此理论基础，目前已有BTK抑制剂选择MCD亚型DLBCL作为目标适应证进行开发。

### 2.2 从“罕见”到“非罕见”

另一方面，随着对疾病认识的不断发展，对肿瘤的认识逐步由组织细胞学向分子学层面发展，因此伴有特定突变的多个罕见瘤种，又可能共同组合成一组非罕见的泛肿瘤。

拉罗替尼（Larotrectinib, LOXO-101）是针对于*NTRK1*/*NTRK2*/*NTRK3*融合基因的靶向治疗药物；在临床试验中，共计入组了17类肿瘤患者，结果显示，对于年龄为4个月至76岁NTRK阳性的各类实体瘤患者，拉罗替尼治疗的整体缓解率为75%，且患者的缓解持久，中位缓解持续时间为49.3个月，中位无进展生存期为35.4个月^[9]^。2018年11月26日美国FDA加速批准泛癌种靶向药拉罗替尼上市，拉罗替尼也成为全球首款广谱靶向药。

目前全球已有5个不限瘤种的抗肿瘤药物上市：①帕博利珠单抗：批准时间：2017年5月，适应证：具有微卫星不稳定（microsatellite instability high, MSI-H）或错配修复缺陷（deficiency of mismatch repair, dMMR）的不可切除实体瘤的成人及儿童患者；②拉罗替尼：批准时间：2018年11月，适应证：*NTRK*融合的成人或儿童实体瘤患者；③恩曲替尼：批准时间：2019年8月，适应证：治疗成人和儿童患者*NTRK*融合阳性、初始治疗后局部晚期或转移性实体肿瘤进展或无标准治疗方案的实体瘤患者以及ROS1阳性非小细胞肺癌患者；④Selpercatinib：批准时间：2020年5月，适应证：晚期*RET*融合阳性非小细胞肺癌、*RET*突变型甲状腺髓样癌和*RET*融合阳性甲状腺癌；⑤多斯塔利单抗：批准时间：2021年8月，适应证：dMMR的复发或晚期实体瘤成年患者。

总之，随着基础研究的发展，根据生物标记物细分人群，可以使我们精准地发现与药物机制更匹配的患病人群，另辟蹊径在“非罕见”中寻找“罕见”，既契合患者需求，又有利于实现药物的差异化研发；反之，通过基于特定的基因突变和生物标记物类型角度定义肿瘤，而非限定于肿瘤的组织学来源，也可能将某种罕见突变的人群扩大，从“罕见”变为“非罕见”，从而减轻临床试验受试者招募困难，降低药物开发难度。

## 3 研发策略的思考

由于罕见肿瘤单个病种的患者人数少、入组困难导致药物临床研发难度大；另一方面，通常这类肿瘤的基础研究较高发肿瘤不足，对疾病本身的认知不足，进一步加剧了药物的研发难度。

罕见肿瘤是罕见病与肿瘤的交集，其药物研发既要满足肿瘤药物研发的要求，又要适应于罕见疾病药物研发的特点。药物研发的难点与药物研发的科学性之间，也并非“不可兼得”的对立关系。罕见肿瘤可参考罕见病药物研发思路^[10]^，在遵循一般药物研发规律的基础上，密切结合罕见肿瘤自身特点，加强临床研究前的机制研究，结合临床实验中的数据以及上市后的数据，综合建立证据链，共同支持药物的获益风险评估；在确保严谨科学的基础上，采用更为灵活的设计，最终在不损失科学性的基础上，实现罕见肿瘤药物的合理、高效研发。

除科学的临床试验设计之外，合理的研发策略也可使得临床研发“事半功倍”。对于罕见肿瘤的药物研发，预先通盘设计临床开发计划也非常重要。与其他罕见病不同，治疗罕见肿瘤的药物往往也可以治疗其他肿瘤，因此，对于药物的探索，可以通过非罕见肿瘤实现，当不同适应证之间有相似的作用机制或相关联的生物标志物时，通过非罕见肿瘤适应证的暴露-效应关系为罕见肿瘤的剂量选择提供支持，这在一定程度上降低了研发难度，但也对临床开发计划的设计提出了要求：当开展罕见疾病药物研发工作时，应对研发计划进行全盘考虑。根据上市的先后顺序，罕见肿瘤药物通常可分为两种情形。

### 3.1 以非罕见肿瘤作为首个适应证上市

此种情况一般会先在非罕见瘤种中对靶点机制、有效安全性进行确证；在对药物充分研究的基础上，进一步根据机制特点，有针对性地开发罕见肿瘤适应证；由于罕见肿瘤中可获得的临床试验数据有限，因此可将罕见肿瘤与非罕见肿瘤的临床数据相结合，共同形成完整的证据链，以支持该药物在罕见肿瘤中的风险获益评估。

恶性黑色素瘤的死亡率仅次于肺癌。在中国，约25%的晚期恶性黑色素瘤患者可检测出*BRAF*驱动基因突变，其中90%以上为V600型。BRAF抑制剂的出现，大大改善了恶性黑色素瘤的预后。BRIM-3研究结果显示，相比传统标准治疗方案达卡巴嗪，维莫非尼的中位OS延长了3.9个月（13.6个月 *vs* 9.7个月），降低了30%的死亡风险（HR=0.70, *P*=0.000, 8）。2011年维莫非尼经美国FDA批准上市。

然而*BRAF* V600E突变并非恶性黑色素瘤所特有。Erdheim-Chester病（Erdheim Chester disease, ECD）是一种非常罕见的、全身性非朗格罕组织细胞增多性疾病，属于血液肿瘤疾病，发病率极低，2018年5月11日，该疾病被列入国家卫生健康委员会等5部门联合制定的《第一批罕见病目录》。大约54%的ECD患者有*BRAF* V600突变。在维莫非尼开展的一项开放标签、多中心、单臂、多队列研究中，入组了22例ECD患者，其中15例患者（68.2%）既往接受过系统治疗，中位随访时间为26.6个月（3.0个月-44.3个月），整体缓解率为54.5%，其中1例患者（4.5%）达到完全缓解。中位持续缓解时间（duration of response, DOR）未达到^[11]^。基于上述结果，维莫非尼于2017年获得了美国FDA的完全批准，适用于伴有*BRAF* V600突变的ECD患者。

维莫非尼的案例提供了一种思路：当疾病致病机制、药物作用机制清晰，并且药物的疗效在具有相似致病机制的疾病中得到证实时，虽然在罕见肿瘤中难以开展大样本量对照研究，但结合先前的研究结果和证据，可以与罕见肿瘤中有限的临床试验数据共同确证其在罕见肿瘤中的疗效。

### 3.2 以罕见肿瘤作为首个适应证上市

某些情况下，也可能选择罕见肿瘤作为首个适应证上市。此种情况下，除有效性数据以外，整体安全性数据不足往往会影响药物的获益风险评估。因此，当计划以罕见肿瘤作为首个适应证上市时，应同步在非罕见肿瘤中开展充分的研究，为新药上市提供更多的安全有效性数据。

毛细胞白血病（hairy cell leukemia, HCL）是一种罕见的惰性B细胞恶性肿瘤，每年在美国和欧洲约确诊25, 881例和16, 002例新发病例，占所有非霍奇金淋巴瘤（non-Hodgkin lymphoma, NHL）的约2%。Moxetumomab是抗CD22单抗偶联药物，其开展的单臂研究1053中，共入组80例既往接受过治疗的HCL患者，结果显示，Moxetumomab单药疗效显著，完全缓解率（complete response rate, CRR）达41.3%，33.8%的患者达到CR伴微小残留病（minimal residual disease, MRD）转阴，64例（64/80, 80%）患者获得血液学缓解（hematological response, HR），CR及HR中位持续时间为62.8个月，中位无进展生存期（progression-free survival, PFS）为41.5个月^[12]^。2018年FDA加速批准Moxetumomab用于治疗难治复发HCL。值得注意的是，虽然关键研究1053的样本量为80例，但在递交上市申请时，安全数据共包含165例包括多种血液系统恶性肿瘤成人患者。

罕见肿瘤在临床试验中所积累的数据通常非常有限，因此上市后的数据收集十分重要，此外还需不断完善上市后风险控制计划，加强药物警戒工作，确保药物上市后临床风险的最小化。

## 4 总结

罕见肿瘤发病率低，药物研发存在挑战，这体现在对疾病认识有限、试验入组困难、研发积极性不足等，这也进一步加剧了罕见肿瘤患者的治疗需求。然而，患者的治疗需求，既是药品研发的方向，也为制药企业带来潜在机遇。

基础研究与转化的加强，致病机制研究的深化，以及探索并发现新的肿瘤分子分型，使“罕见肿瘤”成为一种动态的概念：“罕见”瘤种的概念可能随着精准治疗的发展进一步扩大；也可能随着对肿瘤的认识从组织细胞学向分子生物学方向的转变，使某些罕见致病基因变得不再罕见。

罕见肿瘤同时具备罕见病和肿瘤的特征。药物研发中可参考罕见病药物的研发思路^[9, 13]^，同时结合肿瘤疾病的特征，充分利用非罕见瘤种临床试验中的数据，充分利用科学工具、精巧的试验设计，实现对罕见肿瘤药物研发的推进。
